# *Phyllanthus amarus* shoot cultures as a source of biologically active lignans: the influence of selected plant growth regulators

**DOI:** 10.1038/s41598-022-15309-0

**Published:** 2022-07-07

**Authors:** Barbara Sparzak-Stefanowska, Mirosława Krauze-Baranowska

**Affiliations:** Department of Pharmacognosy with Medicinal Plants Garden, Medical University of Gdańsk, Gdańsk, Poland

**Keywords:** Plant biotechnology, Secondary metabolism, Plant biotechnology

## Abstract

This is the first comprehensive study of the influence of plant growth regulators (PGRs) on the development of shoots and accumulation of biologically active lignans—phyllanthin and hypophyllanthin, in the shoot culture of *P. amarus* Schum. & Thonn. (Euphorbiaceae) obtained by direct organogenesis. The following PGRs were included in the experiments—cytokinins: kinetin (Kin), 6-benzylaminopurine (BAP), 2-isopentenyladenine (2iP), 1-phenyl-3-(1,2,3-thiadiazol-5-yl)urea, thidiazuron (TDZ) and auxin, indole-3-butyric acid (IBA) and used at various concentrations. Depending on PGRs and their concentrations, differences in the culture response and lignan accumulation were observed. The highest content of the investigated compounds was found in the shoot culture grown on Murashige and Skoog’s (MS) medium supplemented with Kin 0.25 mg/L. The sum of phyllanthin and hypophyllanthin was  ~ 10 mg/g of dry weight (DW), which was similar or even higher than that in the plant material obtained from natural conditions. The results of the research provide new data on the selection of the optimal growth medium for the production of plant material with a significant level of phyllanthin and hypophyllanthin biosynthesis. The obtained data may also be valuable in designing systems for large-scale cultivation of *P. amarus* shoots with high productivity of hepatoprotective lignans.

## Introduction

Numerous species of the genus *Phyllanthus* (common name—leafflower) (Euphorbiaceae) have been used for millennia in traditional medicine of India, China and South America, mainly in the treatment of liver and urinary tract diseases^1–5^. Currently, many species of leafflowers are the subject of intense pharmacological research, which indicated, among others, their hepatoprotective properties^3,6,7^, antiviral activity against hepatitis B virus (HBV) as well as anti-inflammatory^3,8^, cytotoxic^3–5,9,10^ and antimicrobial activity^11–14^.

*Phyllanthus amarus* is an annual herb naturally occurring in tropical and subtropical regions of Central and South Asia, up to an altitude of 800–1000 m MSL. The species has a long history of use and is an important plant in the Ayurvedic medical system in the treatment of diseases of liver, genitourinary system, kidney and stomach^15,16^. The pharmacological effects of this plant are attributed to a wide variety of bioactive metabolites, especially lignans (e.g. phyllanthin and hypophyllanthin) (Supplementary Fig. 1), but also tannins, flavonoids, triterpenes, sterols and alkaloids^17–19^. A number of studies of lignans isolated from *Phyllanthus* species, including *P. amarus*, confirm their antiviral and protective activity against the liver cells, and indicate their participation in the anti-inflammatory and cytotoxic effect of medicinal plant materials obtained from leafflowers^2,3,20,21^.

For medicinal purposes, *P. amarus* is obtained from natural conditions and the species is not commonly cultivated. This is a threat that could lead to the extinction of the species. *P. amarus* rarely survives in dry environments or very low temperatures but tolerates waterlogging. Its growing season is short, including the monsoons, from July to October. Each plant produces 50–150 seeds, and the optimal temperature for their germination is 20–35 °C^15,16,22^. In the viability test the maximum number of sinking seeds (58%) was observed for seeds collected from the shade-dried plant between 0 and 3 days^22^. The germinability of natural *P. amarus* seeds is low (29%) and their germination rate drops drastically over time^22^. In order to produce good germinable seeds, it is very important to collect and store them properly (storage at 4 °C without moisture guarantees the viability of seeds up to 12 months). Due to poor germinability and sensitivity to temperature and humidity, the productivity of *P. amarus* is low. Moreover, the plant also requires a high level of calcium fertilization, which further reduces their yield^23,24^. The influence of variable environmental conditions on the level of biosynthesis of biologically active compounds is also significant^25,26^.

Plant in vitro cultures, guarantee isolation from the changing external environment and at least partially, eliminate the above-mentioned problems related to the cultivation of *P. amarus*. As with other types of in vitro plant cultures, in vitro shoot cultures can also be used to produce of biologically active secondary metabolites. Since in vitro shoot cultures preserve the tissue differentiation of the parent plant, they are often capable of biosynthesis of secondary metabolites that are absent in unorganized cell suspensions or callus culture^27^. Recent studies on lignans accumulation in callus cultures of leafflowers support this thesis. The determined content of lignans in the callus biomasses of *Phyllanthus* species is low (at the level of µg/g of DW) and does not reach the lignans concentration in the intact plant^28–30^. In some cases, the callus tissue does not biosynthesize these compounds at all^31^. This confirms the fact, that morphological differentiation is necessary for the biosynthesis of some secondary metabolites^30,32^. Plant material with a high lignan content is valuable because not all *Phyllanthus* species biosynthesize these compounds^4,33^. In our screening test of 5 *Phyllanthus* species only in *P. amarus* we found the presence of lignans and therefore this species was selected for further research.

Previous studies on *P. amarus *in vitro shoot cultures usually concern the influence on the development of the culture of single cytokinins and auxins^34–39^. There are no detailed studies of the influence of particular PGRs on the level of lignan biosynthesis in in vitro shoot cultures of *Phyllanthus* species. Therefore, the subject of our research was a comprehensive analysis of the influence of the most commonly used PGRs (BAP, Kin, TDZ, 2iP and IBA) on the development of *P. amarus* shoot culture obtained as a result of direct organogenesis and also on the accumulation of biologically active lignans—phyllanthin and hypophyllanthin.

## Materials and methods

All the methods were performed in accordance with relevant guidelines and regulations.

### Culture media and growth conditions

All aspects of preparation of culture media and growth conditions were described previously^14,20,40^.

### Plant material and explant preparation

The seeds used for the establishment of in vitro cultures of *Phyllanthus amarus Schum. & Thonn.* (*Euphorbiaceae*) were obtained from the Royal Botanical Garden in Brussels (Belgium) in 2009.

After seeds germination the explants were moved to the Murashige and Skoog (MS) medium^41^ without plant growth regulators. The shoots were subcultured in 5-weeks intervals. To analyze the content of lignans shoots without roots were used. The collected plant material was lyophilized and pulverized.

Additionally in preliminary studies concerning the presence of lignans in different *Phyllanthus* species the following shoot cultures were included—*P. multiflorus* (SH_0_), *P. glaucus*—MS IBA 0.5 mg/L, BAP 0.5 mg/L, *P. juglandifolius* and *P. grandifolius* (both MS BAP 1.0 mg/L, T 0.2 mg/L).

### Influence of PGRs on shoot growth and accumulation of secondary metabolites

The explants of *P. amarus* (the nodal explants grown on MS_0_ medium) were placed onto MS medium enriched with PRGs—(a) single cytokinins – BAP, Kin, 2iP (0.25, 0.5, 1.0, 2.0 mg/L) and TDZ (0.05, 0.1, 0.2, 0.5 mg/L), (b) single auxin—IBA (0.25, 0.5, 1.0, 2.0 mg/L), (c) a mixture of 2iP (0.25, 0.5, 1.0, 2.0 mg/L) and IBA (0.5 mg/L) and (d) mixture of 2iP (0.25, 0.5, 1.0, 2.0 mg/L) and BAP or K (1.0 mg/mL) or TDZ (0.05 mg/L). The following features—shoots length, proliferation (number of new shoots/explant), roots development (number and length), % of rooting explants, the presence and size of callus tissue, and the content of phyllanthin and hypophyllanthin were measured after 35 days of growing. Shoots devoid of roots and callus were used for lignan content analysis. The collected plant material was lyophilized and pulverized. In all experiments, plant material cultivated on the medium without PGRs (MS_0_) was used as control.

### Phytochemical analysis

#### Chemicals

Methanol were obtained from P.O.Ch. (Gliwice, Poland). Acetonitrile was obtained from Merck (Darmstadt, Germany). Phyllanthin and hypophyllanthin standards were purchased from ChromaDex (USA), and the structures are given in Supplementary Fig. 1.

### Analysis of lignans

#### Preparation of extracts

Dried and pulverized plant material (0.5 g) was extracted with methanol in ultrasonic bath (3 × 50 mL, 3 × 30 min) at temperature 50 °C. The methanol extract was evaporated under reduced pressure to a dry residue, which was then re-dissolved in methanol (10 mL).

##### HPLC–DAD-ESI–MS

Analysis was performed on a Shimadzu (Japan) HPLC–DAD-ESI/MS Prominence system equipped with solvent degasser (DGU20A_5_), column thermostat CTO-20AC), autosampler (SIL-20ACXR), pump (LC-20AD), photo diode array detector (SPD-M20A), mass spectrometer (LCMS-2020) (single quadrupole) in the positive electrospray ionization (ESI+) mode, in the mass range m/z 100–450. The optimized ionization parameters were as follows: electrospray voltage 1.2 kV, source temperature 250 °C, nebulizing gas flow 1.5 L/min, heater gas 200 °C, drying gas flow 10 L/min. The system was operated with LabSolution software.

Analyses were performed on Chromolith Performance RP-18E (100–4.6) (Merck, Darmstadt, Germany) at 25 °C. Mobile phase A was acetonitrile and mobile phase B was water. The following linear gradient elution was used: 0–20 min from 40 to 50% mobile phase A. The sample injection volume was 10 μL and the flow rate was 1 mL/min. Chromatograms were recorded at λ—280 nm.

The HPLC method was validated in terms of selectivity, linearity, precision, repeatability, intra- and inter-day precision, LOD, LOQ, and recovery according to the method described earlier^42^. Phyllanthin and hypophyllanthin standard stock solutions (1 mg/mL) were prepared in methanol. For quantitative analysis, the stock solutions was diluted to five different working solutions having concentrations from 250 to 5 µg/mL (250; 125; 75; 25; 5 µg/mL). Standard solutions of phyllanthin and hypophyllanthin at concentration 125 µg/mL were used to establish repeatability, intra- and inter-day precision.

The identification of phyllanthin and hypophyllanthin was carried out by comparison of their retention times, UV spectra and *m*/*z* values of molecular ions with obtained for the standard compounds.

### Analysis of securinega-type alkaloids, flavan-3-ol derivatives and β-sitosterol

Analysis of alkaloids, flavan-3-ol derivatives and β-siterols were performed according to previously established methods^42–44^.

### Total tannins evaluation

Evaluation was performed according to method described in European Pharmacopeia^45^.

### Statistical analysis

Statistical analysis was performed as described earlier^14^.

The results of the quantitative analysis are the mean of 3 trials ± SD. The results of the PGR influence on the development of *P. amarus* shoot culture are the means of ≥ 50 trials ± SD.

## Results

### Phytochemical analysis

The following shoot cultures of leafflower species were included in the screening for lignans—*P. amarus*, *P. multiflorus*, *P. glaucus*, *P. juglandifolius* and *P. grandifolius*. *P. amarus* was the only species that accumulated the analyzed compounds.

**Figure 1:**
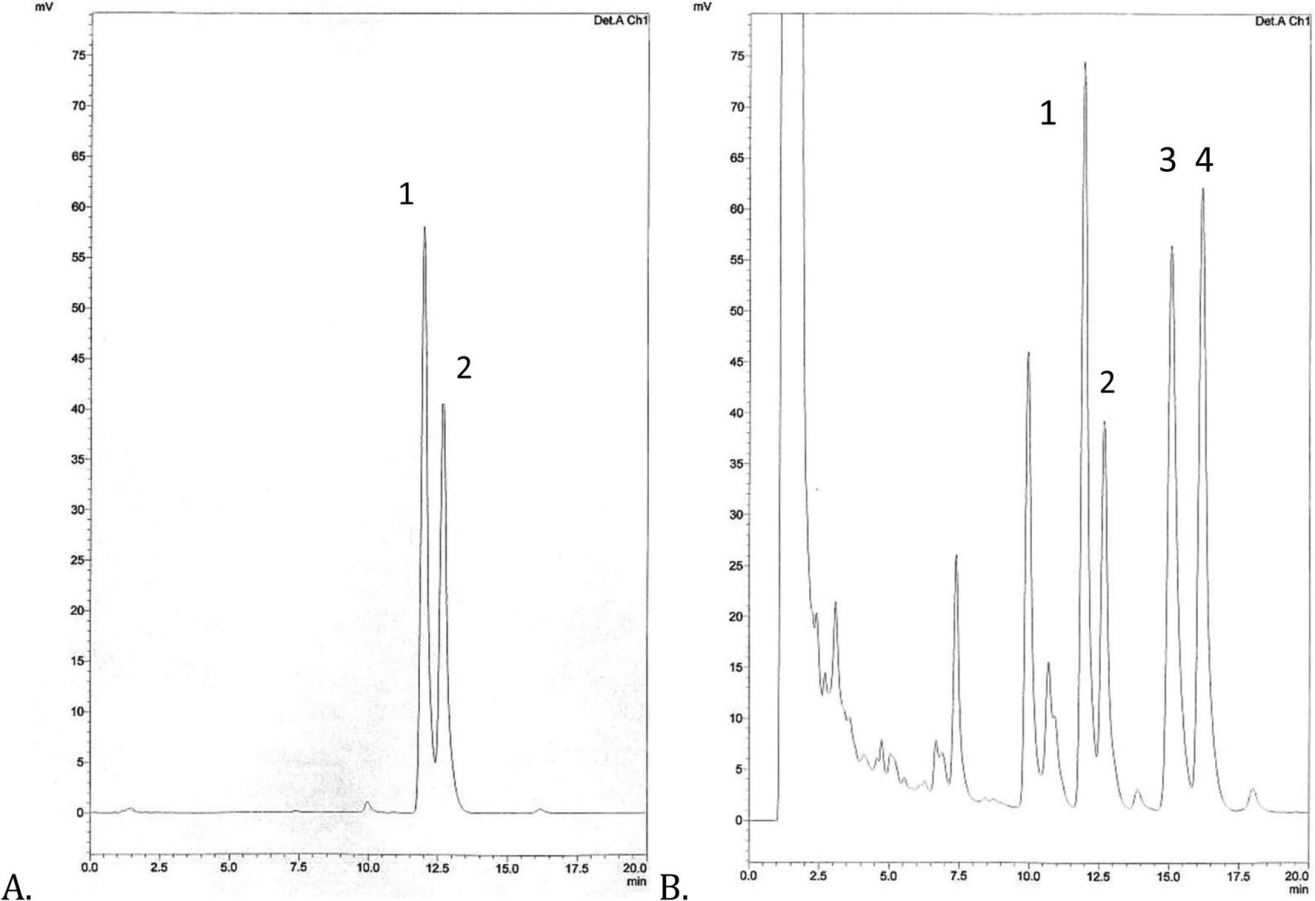
HPLC chromatograms of lignan fraction from the shoot culture of of *P. amarus* harvested on solid MS_0_ medium (**B**), and standard compounds (**A**): 1—phyllanthin, 2—hypophyllanthin, 3—nirtetralin, 4—niranthin. Monolithic column HPLC Chromolith performance RP-18E 100–4.6, T = 25 °C, 0 min—40% B, 20 min—50% B, A—H_2_0, B—ACN, flowrate: 1 mL/min, detection: UV at λ—280 nm.

The separation of lignan complex from *P. amarus* shoots was carried out by a use of HPLC–DAD-ESI–MS method and the following compounds were identified—phyllantin, hypophyllantin, nirantin and nirtetralin. Two lignans were identified by comparison with standard compounds, namely phyllantin (t_R_ 12.2 min) and hypophyllantin (t_R_ 12.8 min) (Fig. 1, Table 1). Peaks eluted at t_R_ 14.9 min and t_R_ 16.0 min were assigned to nirtetralin and nirantin based on data from their ESI–MS spectra and compared with literature data^18,29^ (Fig. 1, Table 1).

**Table 1:** Retention time (HPLC–UV), UV spectra, molecular weight and molecular ions of analyzed compounds.

Compound	Molecular weight	t_R_	UV_max_	Molecular ions	
				[M + H]^+^	[M + Na]^+^
(1) Phyllanthin	418	12.2	229.279	419	441
(2) Hypophyllanthin	430	12.8	235.278	431	453
(3) Nirtetralin	430	14.9	234.281	431	453
(4) Niranthin	432	16.0	232.279	433	455

The developed method is precise, reproducible and linear in the range from 5 to 250 µg/mL for hypophyllantin and also phyllantin. The limit of detection (S/N = 3) and the limit of quantification (S/N = 10) for both lignans are given in Table 2. The mean recovery for phyllanthin was 105.01% and for phyllantin 106.31% (Table 3).

**Table 2:** The validation data for the determination of phyllanthin and hypophyllanthin by elaborated HPLC–UV method.

Validation parameters	Phyllanthin	Hypophyllanthin
Linearity (linear regression equation, regression coefficient)	y = 0.000123995x + 0.295; r = 0.9998	y = 0.00016344x + 1.727; r = 0.9996
Limit of detection(LOD) [ng/ml]	105.9	138.6
limit of quantification (LOQ) [ng/ml]	353	462
Intra-day precission [RSD, %]	0.38	0.17
Inter-day [RSD, %]	0.46	0.48
repeatability [RSD, %]	2.98	2.95

**Table 3:** The recovery of phyllanthin and hypophyllanthin by elaborated HPLC–UV method (n = 3).

Amount present in analysed sample (mg)	Amount added to the sample(mg)	Amount found (mg)	Recovery ± SD. (%)	Mean recovery (%)
**Phyllanthin**				
1.404	0.702	2.205 ± 0.04	104.66 ± 1.74	105.01
	1.404	3.024 ± 0.02	107.69 ± 0.81	
	1.755	3.244 ± 0.00	102.69 ± 0.00	
**Hypophyllanthin**				
1.083	0.542	1.720 ± 0.02	105.82 ± 1.08	106.31
	1.083	2.360 ± 0.01	108.96 ± 0.53	
	1.354	2.538 ± 0.02	104.15 ± 1.28	

Shoots of *P. amarus* cultured on MS_0_ medium were tested for the content of alkaloids, flavan-3-ol derivatives, sterols and triterpenes, in addition to lignans, by methods previously established^42–44^. Among the flavan-3-ol derivatives, the presence of four compound was found—(−)-epicatechin (0.18 ± 0.02 mg/g DW), (+)-catechin (0.33 ± 0.01 mg/g DW), (−)-epigallocatechin (0.36 ± 0.01 mg/g DW), and (−)-gallocatechin (0.41 ± 0.01 mg/g DW). Additionally, the presence of β-sitosterol was detected (4.55 ± 0.23 mg/g d. w.). No securinega-type alkaloids were found. The total tannin content was 1.67%.

### The influence of PGRs on the shoot culture of P. amarus

**Figure 2:**
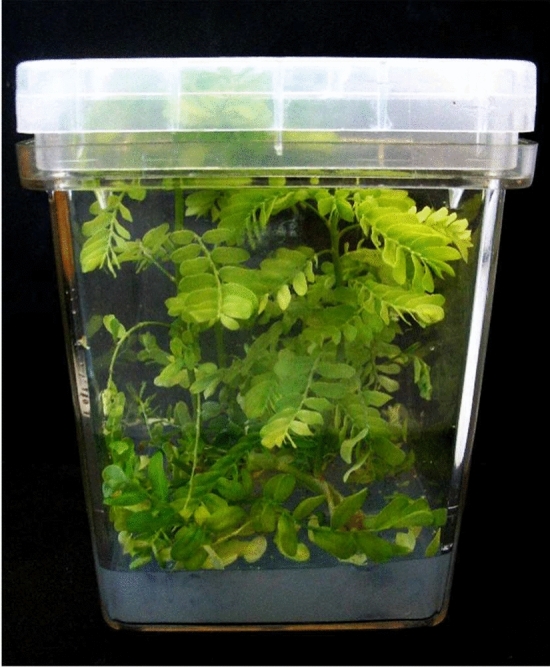
Shoot culture of *P. amarus* (MS_0_).

The following PGRs were used to study the effects of single cytokinins on shoot culture development of *P. amarus*: 2iP, BAP, and Kin (0.25–2.0 mg/L) and TDZ (0.05–0.5 mg/L). Shoots grown in MS0 medium were used as control. They reached an average length of 8 cm and showed no proliferation or callus induction. 100% of the explants rooted spontaneously (Table 4, Fig. 2).

**Table 4:** Effects of single cytokinins on the growth and proliferation of *P. amarus* shoot culture.

Plant growth regulators (mg/L)	Proliferation rate (mean ± SD)	Shoot lenght (cm) (mean ± SD)	Root length (cm) (mean ± SD)	Shoot number/explant (mean ± SD)	% rooting plantlets	Callus [mm] (mean ± SD)
Control (MS_0_)	1.0 ± 0.00^a^	7.97 ± 1.08^a^	3.84 ± 0.90^a^	9.44 ± 2.57^a^	100	–
2iP 0.25	1.42 ± 0.88^b^	5.94 ± 2.08^b^	3.44 ± 1.11^a^	10.00 ± 3.83^a^	100	2.63 ± 0.73
2iP 0.5	1.62 ± 1.06^b^	6.22 ± 1.83^b^	3.40 ± 1.18^a^	7.07 ± 3.91^b^	96.66	4.18 ± 1.38^a^
2iP 1.0	1.58 ± 0.86^b^	5.28 ± 1.79^b^	2.77 ± 1.36^a^	6.15 ± 3.41^b^	91.53	4.64 ± 1.77^a^
2iP 2.0	1.76 ± 1.03^b^	4.33 ± 1.30^c^	1.97 ± 1.51^b^	4.40 ± 3.14^c^	82.76	4.90 ± 1.88^a^
BAP 0.25	1.55 ± 0.78^b^	3.07 ± 1.14^c^	1.25 ± 1.07^c^	3.80 ± 3.52^c^	80.00	7.42 ± 2.98^b^
BAP 0.5	1.70 ± 1.07^b^	2.53 ± 0.95^d^	0.72 ± 0.84^c^	1.78 ± 2.52^e^	57.50	9.68 ± 3.53^d^
BAP 1.0	1.26 ± 0.51^a^	2.49 ± 1.00^d^	0.28 ± 0.44^d^	0.89 ± 1.55^f^	37.14	11.11 ± 4.60^d^
BAP 2.0	1.30 ± 0.73^a^	1.84 ± 0.88^d^	0.18 ± 0.51^d^	0.43 ± 1.43^f^	15.00	10.53 ± 4.39^d^
Kin 0.25	1.98 ± 1.48^c^	5.06 ± 1.78^b^	3.32 ± 1.91^a^	7.30 ± 4.66^b^	94.00	4.46 ± 1.69^a^
Kin 0.5	2.86 ± 1.34^d^	4.52 ± 1.58^c^	2.42 ± 1.19^b^	7.46 ± 4.89^b^	92.00	4.48 ± 1.94^a^
Kin 1.0	2.59 ± 1.38^d^	5.55 ± 1.91^b^	2.83 ± 1.33^a^	9.31 ± 4.84^a^	99.6	4.47 ± 1.88^a^
Kin 2.0	2.75 ± 1.45^d^	5.62 ± 2.12^b^	2.78 ± 1.42^a^	9.86 ± 5.32^a^	99.80	5.35 ± 2.58^c^
TDZ 0.05	1.00 ± 0.00^a^	1.87 ± 1.04^d^	–	–	–	17.20 ± 6.38
TDZ 0.1	1.00 ± 0.00^a^	1.80 ± 0.64^d^	–	–	–	11.40 ± 5.53^de^
TDZ 0.2	1.00 ± 0.00^a^	1.99 ± 1.04^d^	–	–	–	6.15 ± 3.24^bc^
TDZ 0.5	–	–	–	–	–	–

The results are the arithmetic means of ≥ 50 trials ± SD. The values ​​in each column marked with different letters (a, b, c…) indicate statistically significant differences (p < 0.05; Tukey's RIR test).

**Figure 3:**
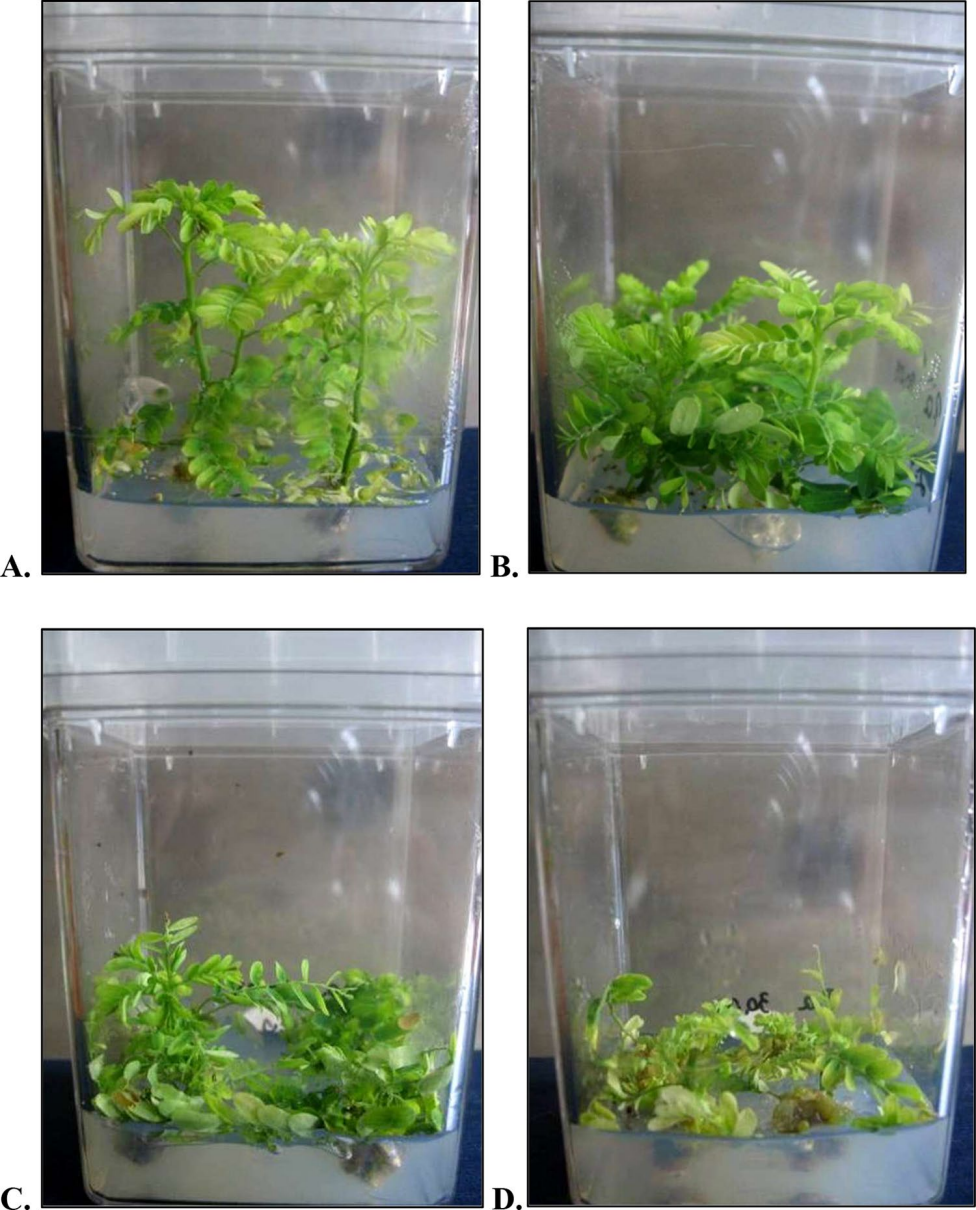
Shoot culture of *P. amarus* (**A**)—MS 1.0 mg/L 2iP, (**B**)—MS 1.0 mg/L Kin, (**C**)—MS 1.0 mg/L BAP, (**D**)—MS 0.1 mg/L TDZ .

The cytokinins BAP, Kin, and 2iP affected shoot proliferation. The highest number of shoots/explant (1.98–2.86) was obtained with 0.5–2.0 mg/L Kin. The other cytokinins stimulated from 1.26 to 1.76 shoots/explant (Table 4). There was a statistically significant reduction in shoot length compared with the control. The strongest effect was observed for TDZ, which at concentrations ranging from 0.05 to 0.20 mg/L strongly inhibited (statistically significant) the development of *P. amarus* shoot culture, yielding shoots less than 2 cm in length, and at the highest concentration, dying of explants was observed (Table 4, Fig. 3.).

Regardless of the type of cytokinin, simultaneous root development and callus formation were observed at the explant cut-off site. As the concentrations of 2iP, Kin and BAP increased, root length and root number decreased. BAP (0.25–2.0 mg/L) showed a stronger inhibitory effect on rhizogenesis (roots length—1.25 to 0.18 cm) compared to 2iP (3.44–1.97 cm) and Kin (3.32–2.78 cm) (Table 4). Using TDZ (0.05–0.5 mg/L), complete inhibition of rhizogenesis was observed. 2iP (0.25–2.0 mg/L) had the weakest effect on callus formation (2.46–4.90 mm), compared, for example, with BAP (from 7.42 to 11.11 mm) or TDZ (from 6.15 to 17.2 mm) (Table 4).

The use of 2iP (0.25–2.0 mg/L) in combination with BAP and Kin (1 mg/L) resulted in 1–2 shoots/explant. The inhibitory effect on rhizogenesis and callus formation was stronger with 2iP in combination with BAP (Table 5). On media supplemented with 2iP (0.25–2.0 mg/L) in combination with TDZ, 2–3 shoots were obtained, but they were very short (≤ 0.51 cm) (Table 5) and also strongly thickened, making separation and passage difficult. The shoots were stunted, produced abundant callus, and had highly altered morphology compared to the control.

**Table 5:** Effect of 2iP supplementation in combination with BAP, Kin or TDZ on the development of *P. amarus* shoot culture.

Plant growth regulators (mg/L)	Proliferation rate (mean ± SD)	Shoot lenght (cm) (mean ± SD)	Root length (cm) (mean ± SD)	Shoot number/explant (mean ± SD)	% rooting plantlets	Callus [mm] (mean ± SD)
Control (MS_0_)	1.0 ± 0.00^a^	7.97 ± 1.08^a^	3.84 ± 0.90^a^	9.44 ± 2.57^a^	100	–
BAP 1.0 2iP 0.25	1.43 ± 1.10^b^	3.46 ± 1.34^c^	0.58 ± 0.73^d^	1.84 ± 2.74^e^	47.05	10.73 ± 3.48^d^
BAP 1.0 2iP 0.5	1.42 ± 1.09^b^	3.93 ± 1.41^c^	0.80 ± 0.85^c^	2.68 ± 3.32^d^	58.00	10.46 ± 3.77^d^
BAP 1.0 2iP 1.0	1.22 ± 0.87^a^	4.64 ± 2.07^bc^	0.85 ± 0.96^c^	3.02 ± 4.15^d^	57.14	10.35 ± 3.82^d^
BAP 1.0 2iP 2.0	1.65 ± 1.26^b^	4.02 ± 1.49^c^	0.46 ± 0.57^d^	1.98 ± 2.77^e^	47.06	9.22 ± 4.36^d^
Kin 1.0 2iP 0.25	1.08 ± 0.34^a^	5.14 ± 1.52^b^	1.71 ± 1.21^b^	6.57 ± 5.14^b^	88.24	5.90 ± 2.33^c^
Kin 1.0 2iP 0.5	1.27 ± 0.72^a^	4.70 ± 1.29^bc^	1.21 ± 0.95^c^	5.25 ± 4.55^c^	86.54	5.38 ± 2.28^c^
Kin 1.0 2iP 1.0	1.24 ± 0.74^a^	4.24 ± 1.06^c^	0.71 ± 0.57^c^	3.06 ± 3.46^d^	76.00	5.22 ± 2.09^c^
Kin 1.0 2iP 2.0	1.43 ± 0.92^b^	4.31 ± 1.37^c^	0.38 ± 0.51^d^	1.76 ± 2.83^e^	45.10	5.90 ± 2.52^c^
TDZ 0.05 2iP 0.25	2.31 ± 1.21^cd^	0.32 ± 0.09^e^	–	–	–	12.06 ± 4.47^e^
TDZ 0.05 2iP 0.5	2.43 ± 1.29^cd^	0.47 ± 0.36^e^	–	–	–	11.98 ± 6.07^d^
TDZ 0.05 2iP 1.0	2.47 ± 1.57^cd^	0.51 ± 0.39^e^	–	–	–	13.59 ± 6.03^de^
TDZ 0.05 2iP 2.0	2.84 ± 1.68^d^	0.51 ± 0.24^e^	–	–	–	13.65 ± 5.36^e^

The results are the arithmetic means of ≥ 50 trials ± SD The values ​​in each column marked with different letters (a, b, c …) indicate statistically significant differences (p < 0.05; Tukey's RIR test).

In studies on auxin effects, IBA (0.25–2.0 mg/L) was tested—as a single PGRs and in combination with 2iP. IBA (0.25–2.0 mg/L) had no effect on shoot proliferation. At concentrations ranging from 0.25 to 0.5 mg/L it did not affect shoot length, while at concentrations 1.0–2.0 mg/L or in combination with 2iP (1.0 mg/L) shorter shoots were obtained (statistically significant difference compared with the control) (Table 6).

**Table 6:** Effect of 2iP and IBA supplementation on the development of *P. amarus* shoot culture.

Plant growth regulators (mg/L)	Proliferation rate (mean ± SD)	Shoot lenght (cm) (mean ± SD)	Root length (cm) (mean ± SD)	Shoot number/explant (mean ± SD)	% rooting plantlets	Callus [mm] (mean ± SD)
Control (MS_0_)	1.0 ± 0.00^a^	7.97 ± 1.08^a^	3.84 ± 0.90^a^	9.44 ± 2.57^a^	100	–
IBA 0.25	1.0 ± 0.00^a^	6.86 ± 2.51^ab^	2.86 ± 1.61^a^	10.16 ± 6.15^a^	98	–
IBA 0.5	1.0 ± 0.00^a^	7.44 ± 2.99^a^	2.30 ± 1.20^b^	7.82 ± 4.88^b^	98	–
IBA 1.0	1.0 ± 0.00^a^	6.34 ± 2.48^b^	1.61 ± 0.71^b^	7.59 ± 5.31^b^	100	–
IBA 2.0	1.0 ± 0.00^a^	4.89 ± 2.14^b^	1.04 ± 0.28^c^	7.03 ± 3.92^e^	97	–
2iP 0.25 IBA 0.5	1.07 ± 0.25^a^	6.31 ± 2.87^b^	2.02 ± 0.69^b^	13.15 ± 7.93^g^	100	5.96 ± 1.40^c^
2iP 0.5 IBA 0.5	1.16 ± 0.47^a^	5.87 ± 2.08^b^	1.86 ± 0.85^b^	12.22 ± 5.47^g^	100	6.30 ± 2.07^c^
2iP 1.0 IBA 0.5	1.24 ± 0.57^a^	5.27 ± 2.90^b^	1.83 ± 0.91^b^	10.71 ± 5.21^a^	100	7.73 ± 2.71^b^
2iP 2.0 IBA 0.5	1.42 ± 0.60^b^	4.90 ± 3.02^b^	1.83 ± 1.06^b^	9.75 ± 4.72^a^	100	7.97 ± 2.02^b^

The results are the arithmetic means of ≥ 50 trials ± SD The values ​​in each column marked with different letters (a, b, c …) indicate statistically significant differences (p < 0.05; Tukey's RIR test).

As IBA concentration increased, root length and number of roots/explant decreased. A similar response of explants was observed using IBA (1.0 g/L) in combination with 2iP, but statistical differences between the values obtained for the above-mentioned parameters were weakly marked (Table 6). On media supplemented with IBA (0.5–2.0 mg/L) alone and in combination with 2iP, the rooting response ranged from 98 to 100% (Table 6).

IBA (0.5–2.0 mg/L) did not induce callus formation, however in combination with 2iP (1.0 mg/L) callus formation was more intense, than if 2iP was used alone (0.25–2.0 mg/L) (statistically significant difference) (Tables 5 and 6).

### The influence of PGRs on the biosynthesis of lignans in the shoot culture of P. amarus

**Figure 4:**
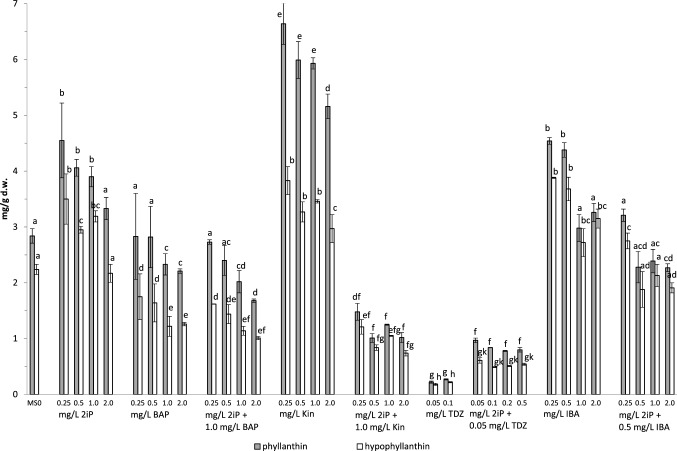
Effect of PGRs on the accumulation of lignans in *P. amarus* shoot culture. Different letters indicate significant differences between means (p < 0.05). n = 3 ± SD.

The study showed a statistically significant effect of PGRs on the accumulation of phyllanthin and hypophyllanthin, compared to the control, which contained 2.87 mg/g DW and 2.24 mg/g DW, respectively (Fig. 4, Supplementary Tables 1, 2, 3).

The highest increase was shown for Kin (0.25–2.0 mg/L) and depending on its concentration it was 5.16–6.64 mg/g DW and 2.97–3.89 mg/g DW for phyllanthin and hypophyllanthin, respectively (2.3-fold and 1.7-fold increase compared to control) (Fig. 4). On medium supplemented with 2iP (0.25–2.0 mg/L), the concentration of phyllanthin and hypophyllanthin in biomasses was 3.33–4.55 mg/g DW and 2.17–3.50 mg/g DW, respectively (Fig. 4). A decrease in lignan concentration with increasing cytokinin concentration was observed for both PGRs, with a statistically significant difference occurring only at the highest Kin and 2iP concentration (2.0 mg/L) (Fig. 4, Supplementary Table 1).

The phyllanthin content in biomasses grown on media with the addition of 0.25–0.5 mg/L BAP was similar to the control sample (~ 2.83 mg/g DW) (Fig. 4, Supplementary Table 1). Higher BAP concentrations (1.0–2.0 mg/L) resulted in a statistically significant decrease in phyllanthin accumulation (2.21–2.33 mg/g DW). Hypophyllanthin content was statistically lower than in the control (Fig. 4, Supplementary Table 1).

The addition of TDZ (0.05–0.5 mg/L) to the medium caused a statistically significant, more than tenfold decrease in phyllanthin and hypophyllanthin content compared to the control, depending on the concentration of TDZ in the medium (Fig. 4, Supplementary Table 1).

There were variable effects of medium supplementation with 2iP (0.25–2.0 mg/L) in combination with other PGRs on lignan biosynthesis level. 2iP in combination with Kin (1.0 mg/L) reduced the accumulation of phyllanthin and hypophyllanthin compared with supplementation with a single PGR (Fig. 4, Supplementary Tables 1, 2). There were no significant differences in lignan content in biomasses obtained on media supplemented with BAP alone or together with 2iP. However, application of 2iP (0.25–2.0 mg/L) with TDZ increased the concentration of lignans, compared to supplementation with TDZ alone (Fig. 4, Supplementary Tables 1, 2).

Supplementation with 0.25–0.5 mg/L IBA resulted in a statistically significant increase of phyllanthin concentration (4.34–4.58 mg/g DW), which decreased with IBA 1.0–2.0 mg/L to that of the control. The concentration of hypophyllanthin (2.72–3.88 mg/g DW) was higher compared to the control over the full range of IBA concentrations. In biomasses grown on media supplemented with IBA (1.0 mg/L) and 2iP (0.25–2.0 mg/L) compositions, the content of phyllanthin and hypophyllanthin was lower compared to supplementation with single PGRs (Fig. 4, Supplementary Tables 1, 3).

## Discussion

Due to the growing demand for plant raw material from *P. amarus* and its biologically active compounds, it seems necessary to develop alternative breeding methods of the species. The cultivation of *P. amarus* in natural conditions requires appropriate humidity and composition of soil substrate. The level of lignan biosynthesis in *P. amarus* is also influenced by many environmental factors such as average rainfall and temperature, duration of snow cover, soil type, radiation intensity and the length of the growing season, related to the geographical altitude^25,26^. The in vitro conditions partially eliminate the aforementioned limitations and variable factors and can provide high-quality plant material, that will be a source of plantlets that will later grow ex vitro or will allow to obtain biomass with a high content of particular compounds.

There are relatively many studies on the multiplication of *P. amarus *in vitro but the results of individual studies take into account different concentrations of particular PGRs, different explants and culture conditions, hence the fragmentary data obtained by various authors are difficult to compare^30,34–36,38,39,46^. Individual authors have been observed very different effects for cytokinins in shoot/explant number and % explant response, even when the same concentrations are used. The proliferation rate for BAP and Kin used separately at the concentration of 0.5 mg/L ranged from 1 to 18 new shoots/explant and 3.5–15 shoots/explant, respectively^34,35,38,39^ and may be due to the different origin of the explants. Ghanti et al. observed, that the largest number of *P. amarus* shoots was regenerated on the medium supplemented with BAP 0.5 mg/L from shoot tip explants (18.3) compared to internodal (12.6) and nodal (6.7) explants^39^. However, it should be emphasized, that even using the same PGR’s in the same concentration and the same type of explants different results can be obtained^37–39^. Interpretation of individual results obtained in the assessment of the effect of cytokinins in combination with auxin is also difficult^34,46^. Hence, in order to make a proper comparison of the influence of individual PGRs on the development of *P. amarus* shoot cultures, it is necessary to observe their effects in experiments conducted simultaneously.

On the basis of results obtained in our research, cytokinins can be classified depending on the influence they exert on the morphology of *P. amarus*. 2iP had little effect on changes in shoots morphology, which especially at lower concentrations (0.25–1.0 mg/L), were very similar to the control (MS_0_) (Figs. 2, 3). A similar effect was observed with kinetin supplementation, obtaining slightly shorter shoots compared to 2iP. BAP, along with an increase in concentration, stimulated stunting and deformation of shoots, while TDZ clearly inhibited the growth of *P. amarus* shoot culture (Table 4, Figs. 2, 3).

Among the cytokinins, the effect of 2iP on leafflowers shoot cultures is relatively poorly understod. Cultivated *P. urinaria* and *P. caroliniensis* on the medium with the addition of 2iP at a concentration of 5 µM gave about 17 shoots/explant and 4–5 shoots/explant, respectively^47,48^. On the other hand, the studies of *P. stipulatus* did not show any effect of 2iP on the propagation^49^. The influence of 2iP on *P. amarus *in vitro cultures has not been analyzed so far. Our research showed that 2iP, both used alone and in combination with other PGRs (TDZ, BAP, Kin, IBA), has little effect on the rate of proliferation (Table 4). However, other cytokinins used in the experiment, including BAP and Kin, which are mentioned as the most effective growth regulators in the propagation of this species, also had a rather weak influence on *P. amarus* shoots proliferation of (Table 4) and this did not confirm some of the literature data^34,37^. Among the tested cytokinins, 2iP slightly inhibited rhizogenesis and only slightly induced callus formation at the base of *P. amarus* shoots (Table 4). A reduction in leaf fall was also observed at the end of the breeding cycle. The process of leaf fall of varying intensity was observed for all PGRs used in these experiments.

In the studies carried out earlier for *P. glaucus*, 2iP also did not promote shoots proliferation and caused a reduction of shoot length, however, unlike *P. amarus* complete inhibition of rhizogenesis was observed under the influence of this PGR. In terms of accumulation of secondary metabolites 2iP had negative influence on the concentration of indolizidine alkaloids—securinin and allosecurinin present in the culture of *P. glaucus* shoot^14^. The results obtained for *P. amarus* and the previously published data on *P. glaucus* shoot cultures^14^ showed significant interspecies diversification in response to the action of individual PGRs, indicating the need for individual selection of the conditions for leafflower species, depending on the planned purpose of the experiments (biomass multiplication or accumulation of secondary metabolites).

Lignans are a structurally diverse group of plant secondary metabolites that are widespread in the kingdom of higher plants. These compounds have dimeric structures formed by a β,β′-linkage between two phenylpropane units with a different degree of oxidation in the side chain and a different substitution on the aromatic moieties^50^. They possess many valuables types of pharmacological activity making them an important source of novel drug candidates and/or leading structural scaffolds used in medicinal chemistry. Biologically active lignans are common in e.g., *Linum*, *Schisandra*, *Sesamum* or *Podophyllum* species^50–52^.

One of the richest dietary sources of lignans are flax seeds. They contain i.a. secoisolariciresinol diglucoside and its aglycone secoisolariciresinol, which are metabolized to mammalian lignans known as enterodiol and enterolactone in the presence of the enzymes of the intestinal microflora. These compounds are functionally similar to estrogens and contribute to a number of human health benefits—they reduce the risk of breast and prostate cancer and improve hyperglycemia^51,53^.

The importance of lignans in the biological activity of *Phyllanthus* species has been the subject of numerous studies, which mainly concern their anti-viral and protective effect on liver cells and their activity in diseases of urinary system^54–58^. Among the lignans most abundant in *P. amarus* phyllanthin and hypophyllanthin are distinguished (Supplementary Fig. 1), as well as niranthine, nirtetralin, and pyltetralin^17,18^. Phyllanthin dominates in the lignan complex. In plant material obtained from natural conditions its content is variable and usually ranges from 3 to 7 mg^18,25,59,60^. The richest source of lignans are leaves of *P. amarus* and it has been shown that for plants growing at sites higher above sea level, phyllanthin concentration can even reach more than 11 mg/g DW. However, due to the small amounts in stems their average concentration in above-ground part was significantly lower (2–3 mg/g DW)^25^. The content of hypophyllanthin in *P. amarus* is usually lower than that of phyllanthin, ranging from 1.8 to 3.2 mg/g DW, respectively^18,19,59,60^.

The lignans identified in the studied culture of *P. amarus* shoots belong to two different types of lignans and show different patterns of fragmentation. Based on the literature data^18,61,62^, in the obtained ESI–MS spectra, apart from the molecular ions [M + Na]^+^ and [M + H]^+^ (Table 1), the presence of fragmentic ions specific to each type of lignans was observed. In the ESI–MS spectra of the aryltetralin-type lignans, namely hypophyllantin and nirtetralin, fragmentic ions were present at m/z 293, which correspond to dimethoxyphenyl groups, as well as the ions at m/z 261. On the other hand, in the ESI–MS spectra of arylbutane type lignans—phyllanthin, niranthin, fragmentic ions which are formed by loss of one [M − CH_3_O^-^]^+^ or two methoxyl groups 369 [M − (2 × CH_3_O + H)]^+^ (Table 1) were present, at m/z 387 and 401 and at m/z 355 and 369, respectively.

The available data on the effect of PGRs on the content of lignans in *P. amarus* cultures in vitro mainly concern callus tissue or regenerated microshoots^28–31^. The studies conducted so far indicate that the callus of the *Phyllanthus* species is a poor source of lignans (lignan content in the range of µg/g DW)^28–30^.

The studies performed by Nitnaware et al.^30^ showed that the highest concentration of lignans was found in the callus culture of *P. amarus* cultivated on the MS medium supplemented with BAP, the lower with TDZ, and the lowest with the Kin, ranging from 4.6 to 42 µg/g DW. Lignans concentration was inversely proportional to cytokinin concentration^30^. The inverse effect of lignan content on auxin (IAA, NAA, 2,4-D) concentration was less marked. The highest content of phyllanthin and hypophyllanthin (0.84 µg/g DW, 0.38 µg/g DW, respectively) was determined in the biomass grown on MS medium supplemented with NAA 2.15 µM and the lowest on MS with the addition of 2.4-D (~ 0.10 µg/g DW, 0.04 µg/g. DW respectively). Simultaneous supplementation with auxins and cytokinins also resulted in a very low concentration of lignans which was at similar to supplementation with cytokinins alone^30^. A study by Nikule^29^ on the *P. tenellus* callus culture showed that the lignan content was higher on NAA; followed by TDZ and IAA (~ 100 to 200 µg/g DW), respectively, while the content in callus cultivated on MS supplemented with IBA, BAP, and Kin (50 µg/g DW) was low^29^. Extremally low concentration of individuals lignans in the callus culture of *P. amarus* and *P. urinaria* was found by Muthusamy et al. (~ 0.1–2.5 µg/g DW). In the study of de Oliveira no lignans were found in the callus culture of *P. amarus*^31^. Nitnaware et al. showed that concentration of phyllanthin and hypophyllanthin in the microshoots regenerated from leaf-derived callus in a presence of TDZ was several times higher (456.4 µg/g and 332.7 µg/g, respectively) than in callus, but it was much lower than in the plant material from the natural habitats^25,59,60^. These results confirmed the thesis that some secondary metabolites morphological differentiation is necessary to obtain a higher yield of secondary metabolites^30,32^.

So far, no comprehensive research has been carried out on the effect of single plant growth regulators on the level of lignan accumulation in *Phyllanthus* shoot cultures obtained by direct organogenesis, which is a desirable method of shoots multiplication that guarantees genetic stability and prevents or reduces the occurrence of somaclonal variation. In the presented studies, it was observed that for individual growth regulators the growth inhibition of cultures was accompanied by a decrease in the concentration of lignans in the biomass. TDZ in concentration 0.05–0.1 mg/L caused a statistically significant, more than tenfold decrease in the level of phyllanthin and hypophyllanthin, compared to the control, and clearly inhibited culture growth, while at concentration of 0.2–0.5 mg/L death of explants was observed. Similar results were observed in the study of the effect of PGRs on the biosynthesis of securinega-type alkaloids in the cultivation of *P. glaucus* shoot—TDZ inhibited shoot growth and decreased the content of alkaloids^14^.

The effect of growth regulators on cultured plant cells include their growth, metabolism and the process differentiation. It is generally believed that PGRs do not react with intermediates of the biosynthetic pathways but appear to alter cytoplasmic conditions for product formation. Elevated levels of cytokinins in the medium affect cell differentiation. The production of metabolites related to such differentiation is expressed/enhanced in culture. The effect of PGRs on the level of secondary metabolite biosynthesis (including lignans) is very variable and difficult to predict. Even within the same species, different results are obtained when cultivating different types of biomass (e.g. shoot culture or callus culture)^63^.

The presented research showed that low Kin concentrations (0.25–0.5 mg/L) can be used to obtain *P. amarus* shoot culture by direct organogenesis with a high content of analyzed lignans (above 10 mg/g DW) (twice as high as in the control sample) (Fig. 4, Supplementary Table 1). Moreover, the study showed that 2iP and IBA (0.25 mg/L) used separately have the potential as PGRs, which significantly increase the level of lignan accumulation compared to the control sample (total content of phyllanthin and hypophyllanthin ~ 8 mg/g DW) (Fig. 4, Supplementary Tables 1, 3). These concentrations are comparable to plant material originated from natural conditions^25,59,60^.

## Conclusion

This is the first comprehensive study on the influence of PGRs on the development of shoots and the accumulation of biologically active lignans—phyllanthin and hypophyllanthin, in the shoot culture of *P. amarus* obtained by direct organogenesis. The obtained data compared the effect of 5 selected plant growth regulators, cytokinins—Kin, BAP, 2iP, TDZ and auxin—IBA used in a different concentrations.

The studies showed that the accumulation of lignans was dependent on the type of PGRs and their concentration in harvesting medium. On the basis of the obtained results, the cytokinins used can be divided depending on the influence they exert on the morphology of *P. amarus*, into those that have a positive effect (Kin, 2iP) and those that slightly (BAP) and significantly limit the growth of culture (TDZ). Growth inhibition was observed to be accompanied by a decrease in lignan biosynthesis and a more than tenfold decrease in phyllanthin and hypophyllanthin was observed with TDZ supplementation, compared to control. The highest content of tested compounds was found in the shoot culture grown on MS medium supplemented with Kin, 2iP or IBA (0.25 mg/L). The content of lignans as the sum of phyllanthin and hypophyllanthin was at the level ~ 8–10 mg/g DW, which is similar or even higher than the content in the plant material collected from natural conditions. Due to the demand for raw plant material, the limited possibilities of obtaining it and due to the low content of these compounds in the biomass obtained so far in in vitro conditions, it is a significant value and achievement of the research carried out.

The research results provide new data facilitating the selection of the optimal culture medium for the production of plant material with a significant level of phyllanthin and hypophyllanthin biosynthesis. The obtained data may also be a starting point for the design of bioreactor systems for large-scale cultivation of *P. amarus* shoots with high productivity of hepatoprotective lignans e.g. using different elicitors.

## Supplementary Information

Supplementary Information.

## Data Availability

The datasets generated and analyzed during the current study are available from the corresponding author on reasonable request.
