# Supplementary material for: Phyllanthus amarus shoot cultures as a source of biologically active lignans: the influence of selected plant growth regulators
Source: Sci Rep. 2022 Jul 7;12:11505. doi: 10.1038/s41598-022-15309-0 (PMC9263152; doi:10.1038/s41598-022-15309-0)
Supplement: Supplementary file 1 — Supplementary Information. [file 41598_2022_15309_MOESM1_ESM.docx]

***Phyllanthus amarus* shoot cultures as a source of biologically active lignans – the influence of selected plant growth regulators**

**Barbara Sparzak-Stefanowska^a^, Mirosława Krauze-Baranowska^a^***

^a^ Department of Pharmacognosy with Medicinal Plants Garden, Medical University of Gdańsk, Gdańsk 80-210, Poland, [bsparzak@gumed.edu.pl](mailto:bsparzak@gumed.edu.pl), krauze@gumed.edu.pl

*Corresponding author. Tel.: +48 58 349 31 60. Al. Gen. J. Hallera 107, 80-416, Gdańsk. e-mail address: krauze@gumed.edu.pl

A.
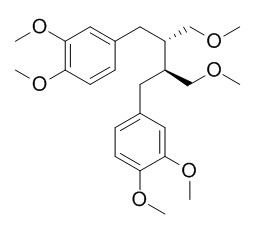
 B.
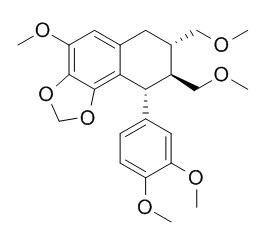


**Supplementary Figure 1.** Structure of phyllanthin (A) and hypopyllanthin (B).

| **Medium** | **Lignan content**  **mg/g DW** | |  |
| --- | --- | --- | --- |
|  | **phyllanthin**  **mg/g DW** | **hypophyllanthin**  **mg/g DW** | |
| **MS_0_ (control)** | **2,87** ± 0,13 ^a^ | **2,24** ± 0,09 ^a^ | |
| **2iP 0,25 mg/L** | **4,55** ± 0,67 ^b^ | **3,50** ± 0,45 ^b^ | |
| **2iP 0,5 mg/L** | **4,06** ± 0,15 ^b^ | **2,95** ± 0,06 ^c^ | |
| **2iP 1,0 mg/L** | **3,90** ± 0,18 ^b^ | **3,19** ± 0,10 ^b, c^ | |
| **2iP 2,0 mg/L** | **3,33** ± 0,20 ^a^ | **2,17** ± 0,16 ^a^ | |
| **BAP 0,25 mg/L** | **2,83** ± 0,77 ^a^ | **1,75** ± 0,41 ^d^ | |
| **BAP 0,5 mg/L** | **2,82** ± 0,55 ^a^ | **1,64** ± 0,34 ^d^ | |
| **BAP 1,0 mg/L** | **2,33** ± 0,19 ^c^ | **1,22** ± 0,18 ^e^ | |
| **BAP 2,0 mg/L** | **2,21** ± 0,04 ^c^ | **1,26** ± 0,03 ^e^ | |
| **Kin 0,25 mg/L** | **6,64** ± 0,37 ^e^ | **3,83** ± 0,25 ^b^ | |
| **Kin 0,5 mg/L** | **5,99** ± 0,33 ^e^ | **3,27** ± 0,18 ^b^ | |
| **Kin 1,0 mg/L** | **5,93** ± 0,10 ^e^ | **3,46** ± 0,03 ^b^ | |
| **Kin 2,0 mg/L** | **5,16** ± 0,22 ^d^ | **2,97** ± 0,25 ^c^ | |
| **TDZ 0,05 mg/L** | **0,22** ± 0,02 ^g^ | **0,18** ± 0,02 ^h^ | |
| **TDZ 0,1 mg/L** | **0,27** ± 0,01 ^g^ | **0,22** ± 0,01 ^h^ | |
| **TDZ 0,2 mg/L** | **-** | **-** | |
| **TDZ 0,5 mg/L** | **-** | **-** | |

**Supplementary Table 1.** Effect of single cytokinins on the accumulation of lignans in *P. amarus* shoot culture. Different letters indicate significant differences between means (p<0.05). n=3 ± SD

| **Medium** | **Lignan content**  **mg/g DW** | | |
| --- | --- | --- | --- |
|  | **phyllanthin**  **mg/g DW** | | **phyllanthin**  **mg/g DW** |
| **MS_0_ (control)** | **2,87** ± 0,13 ^a^ | **2,24 ± 0,09 ^a^** | |
| **BAP 1,0 mg/L**  **2iP 0,25 mg/L** | **2,73** ± 0,04 ^a^ | **1,62 ±** 0,00 ^d^ | |
| **BAP 1,0 mg/L**  **2iP 0,5 mg/L** | **2,40** ± 0,27 ^ac^ | **1,44** ± 0,17 ^de^ | |
| **BAP 1,0 mg/L**  **2iP 1,0 mg/L** | **2,02** ± 0,20 ^cd^ | **1,14** ± 0,08 ^ef^ | |
| **BAP 1,0 mg/L**  **2iP 2,0 mg/L** | **1,68** ± 0,03 ^d^ | **1,01** ± 0,03 ^ef^ | |
| **Kin 1,0 mg/L**  **2iP 0,25 mg/L** | **1,48** ± 0,15 ^df^ | **1,21** ± 0,13 ^ef^ | |
| **Kin 1,0 mg/L**  **2iP 0,5 mg/L** | **1,01** ± 0,08 ^f^ | **0,84** ± 0,05 ^fg^ | |
| **Kin 1,0 mg/L**  **2iP 1,0 mg/L** | **1,25** ± 0,01 ^f^ | **1,05** ± 0,01 ^efg^ | |
| **Kin 1,0 mg/L**  **2iP 2,0 mg/L** | **1,02** ± 0,09 ^f^ | **0,74** ± 0,05 ^fg^ | |
| **TDZ 0,05 mg/L**  **2iP 0,25 mg/L** | **0,97** ± 0,04 ^f^ | **0,61** ± 0,05 ^gk^ | |
| **TDZ 0,05 mg/L**  **2iP 0,5 mg/L** | **0,84** ± 0,00 ^f^ | **0,49** ± 0,01 ^gk^ | |
| **TDZ 0,05 mg/L**  **2iP 1,0 mg/L** | **0,78** ± 0,01 ^f^ | **0,51** ± 0,01 ^gk^ | |
| **TDZ 0,05 mg/L**  **2iP 2,0 mg/L** | **0,80** ± 0,04 ^f^ | **0,54** ± 0,02 ^gk^ | |

**Supplementary Table 2.** Effect of 2iP in combination with other cytokinins on the accumulation of lignans in *P. amarus* shoot culture. Different letters indicate significant differences between means (p<0.05). n=3 ± SD

| **medium** | **Lignan content**  **mg/g DW** | |
| --- | --- | --- |
|  | **phyllanthin**  **mg/g DW** | **hypophyllanthin**  **mg/g DW** |
| **MS_0_ (control)** | **2,87** ± 0,13 ^a^ | **2,24** ± 0,09 ^a^ |
| **IBA 0,25 mg/L** | **4,54** ± 0,06 ^b^ | **3,88** ± 0,01 ^b^ |
| **IBA 0,5 mg/L** | **4,38** ± 0,13 ^b^ | **3,68** ± 0,21 ^b^ |
| **IBA 1,0 mg/L** | **2,98** ± 0,24 ^a^ | **2,72** ± 0,25 ^bc^ |
| **IBA 2,0 mg/L** | **3,26** ± 0,16 ^a^ | **3,15** ± 0,17 ^bc^ |
| **2iP 0,25 mg/L**  **IBA 0,5 mg/L** | **3,21** ± 0,11 ^a^ | **2,75** ± 0,14 ^c^ |
| **2iP 0,5 mg/L**  **IBA 0,5 mg/L** | **2,28** ± 0,28 ^acd^ | **1,88** ± 0,32 ^ad^ |
| **2iP 1,0 mg/L**  **IBA 0,5 mg/L** | **2,39** ± 0,21 ^ac^ | **2,13** ± 0,20 ^a^ |
| **2iP 2,0 mg/L**  **IBA 0,5 mg/L** | **2,27** ± 0,07 ^cd^ | **1,91** ± 0,09 ^ad^ |

**Supplementary Table 3.** Effect of IBA on the accumulation of lignans in *P. amarus* shoot culture. Different letters indicate significant differences between means (p<0.05). n=3 ± SD
